# Mechanotransduction of Piezo1 in the cancer microenvironment: implications for NK cell-based immunotherapy

**DOI:** 10.3389/fonc.2025.1729588

**Published:** 2026-01-13

**Authors:** Xinmu Cui, Binbin Zhang, Jianan Zhao, Huajie Tian

**Affiliations:** 1 Department of Medical Technology, Changchun Medical College, Changchun, China; 2 School of Clinical Medicine, Hangzhou Normal University, The Affiliated Hospital of Hangzhou Normal University, Hangzhou, Zhejiang, China; 3 Department of Cardiovascular Sciences, Temple University, Philadelphia, PA, United States; 4 Department of Hematology, Shuguang Hospital Affiliated to Shanghai University of Traditional Chinese Medicine, Shanghai, China

**Keywords:** cancer immunotherapy, immunotherapy, matrix stiffness, mechanobiology, mechanotransduction, natural killer cells, Piezo1, TME

## Abstract

Natural Killer (NK) cells serve a critical function in antitumor immunity. However, their effectiveness is often hampered by the biomechanical properties of the solid tumor microenvironment (TME), such as the stiffness of the extracellular matrix. This review focuses on the mechanosensitive ion channel Piezo1 and its role in enhancing NK cell function. Studies have shown that tumor cell stiffness, as a key physical cue, directly influences the responsiveness of NK cells. In three-dimensional (3D) matrices, the stiffening of the extracellular matrix (ECM) can activate Piezo1, leading to calcium influx that substantially boosts NK cells’ cytotoxicity and tumor infiltration ability. Remarkably, similar to Yoda1, a specific Piezo1 agonist, short-term Piezo1 activation significantly enhances NK cells’ cytotoxicity and infiltration capacity. Whether such benefits persist under prolonged stimulation without inducing functional exhaustion remains to be determined. Unlike broader review articles that discuss TME biomechanics, this study focuses on uncovering the signal transduction mechanism of the Piezo1–NK cell axis, providing new perspectives and strategies for addressing immunotherapy resistance. This mechanobiology-based framework, through detailed analysis of the Piezo1-NK cell signaling transduction mechanism, is expected to overcome bottlenecks in NK cell immunotherapy. Its application prospects are not limited to the field of oncology but can also be extended to other diseases sensitive to mechanical signals.

## 1 Introduction

The tumor microenvironment (TME) constitutes a unique and sophisticated ecological system, where prominent physical barriers often become key bottlenecks restricting therapeutic efficacy (1). While the academic community has conducted in-depth research on the mechanisms by which biochemical signals such as those mediated by prostaglandin E2 (PGE2) and transforming growth factor-β (TGF-β) regulate tumor immunosuppression, the complex biophysical landscape of solid tumors poses an undeniable challenge to immunotherapy (2, 3). This mechanical stress is a composite of multiple forces, including elevated interstitial fluid pressure that generates compressive forces, altered fluid shear stress in blood and lymphatic vessels, and profound changes in extracellular matrix (ECM) architecture and stiffness. Among these factors, abnormal ECM stiffness has emerged as a particularly well-defined physical cue that modulates natural killer (NK) cell function via Piezo1-dependent mechanotransduction. However, it is crucial to recognize that these physical cues often act in concert; for instance, increased matrix deposition not only heightens stiffness but also elevates solid stress and alters interstitial flow, collectively shaping the immune response. Therefore, while this review focuses on stiffness-mediated Piezo1 signaling, we acknowledge its place within a broader spectrum of mechanobiological inputs that govern NK cell fate (4). For innate defenders like NK cells, the exertion of their antitumor efficacy relies on overcoming these physical barriers; thus, these mechanical obstacles not only serve as major drivers of tumor immune evasion but also represent a critical crux behind the suboptimal efficacy of various cell-based therapies (5). Central to deciphering this mechanical regulation is the mechanosensitive ion channel Piezo1, which can convert mechanical stress into biochemical signals (6). While this review focuses primarily on NK cells, Piezo1’s importance as a core regulator of mechano-immunology is self-evident, and its multifaceted roles in the immune system are particularly prominent (7). For instance, in human T cells, Piezo1 is critical for optimal activation: it enhances T cell receptor (TCR)-mediated proliferation and cytokine secretion by promoting calcium ion (Ca^2+^) influx (8, 9). Additionally, it modulates macrophage polarization and dendritic cell function. Therefore, targeting Piezo1 can orchestrate a multi-dimensional anti-tumor immune response (10, 11). The pleiotropic roles of Piezo1 underscore its central importance in human physiology and disease. Recent studies have revealed that Piezo1 is involved in multiple physiological systems, serving as a key shear stress sensor in the vascular system and playing a role in the regulation of osteoblast differentiation and bone remodeling in the skeletal system. Additionally, its involvement in renal function, pulmonary ventilation, and neurological disorders suggests that Piezo1 is a promising therapeutic target for various diseases, including cancer, cardiovascular conditions, musculoskeletal disorders, and inflammatory diseases, where it may influence the function of NK cells in antitumor activity (4, 12, 13). In NK cells, Piezo1 plays a vital role in enhancing antitumor function: acute activation can trigger Ca^2+^ influx and the Yes-associated protein/Transcriptional co-activator with PDZ-binding motif (YAP/TAZ) signaling pathway, thereby increasing the release of cytotoxic granules and boosting NK cell infiltration by 2- to 3-fold (14). This favorable response is particularly evident in tumors with high collagen density. Remarkably, Piezo1 activation enhances NK cell cytotoxicity and infiltration capacity in short-term settings, and this activation can be achieved either through matrix stiffness or via the agonist Yoda1. Whether this effect persists under prolonged stimulation without inducing functional exhaustion remains to be investigated (14).

Compared with simply activating Piezo1, precision modulation of this mechanosensitive ion channel represents a more promising intervention strategy. Specifically, the combined use of short-acting Piezo1 agonists and ECM-remodeling agents exerts a synergistic effect to “soften” the TME, thereby creating favorable conditions for immune cell infiltration (1, 15). Notably, this strategy offers significant advantages over existing cell therapies. For instance, Chimeric Antigen Receptor T-cell Therapy (CAR-T therapy) faces considerable bottlenecks in solid TME (16): it not only struggles to penetrate dense solid tumor tissues but also has a neurotoxicity incidence of 25% to 40% (17). Conceptually, the integration of rationally designed Piezo1 modulation with Chimeric Antigen Receptor Natural Killer Cell Therapy (CAR-NK) holds potential to mitigate on-target off-tumor toxicities such as neurotoxicity by precisely fine-tuning NK cell activation thresholds (18, 19). However, this combinatorial approach remains hypothetical, as no clinical evidence currently exists to quantify its effects on neurotoxicity incidence. Unlike immune checkpoint inhibitors, which primarily target biochemical suppressive signals in the TME, this Piezo1-centered strategy directly addresses physical barriers, thus filling a critical gap in overcoming treatment resistance (20). In recent years, existing studies have extensively explored the role of biomechanics in the TME, but most focus on general regulatory mechanisms and lack in-depth analysis of the interactions between specific mechanosensors and specific immune cell subsets. However, this review differs: it takes the mechanosensitive ion channel Piezo1 as the core and systematically dissects its regulatory role in NK cell function.

Therefore, this review aims to systematically elucidate how mechanical stress regulates the NK cells’ function in tumor suppression through the core hub of Piezo1, and uncover the intricate molecular mechanisms involved in this process (21). By synthesizing critical evidence from existing studies, we propose innovative targeted therapeutic strategies that offer distinct advantages. We elucidate Piezo1’s role as a “mechanical signal transduction hub” which translates physical cues in TME (such as ECM stiffness) into functional activities of NK cells, including cytotoxicity and infiltration. This approach aims to overcome the physical barriers that currently hinder NK cells’ effectiveness in solid tumors (22). This perspective has not been systematically elaborated in previous studies involving multiple cell types and mechanosensors. Ultimately, the focused analytical framework established in this review is expected to narrow the translational gap between basic research and the development of precision immunotherapies, thus paving the way for more effective and durable solid tumor treatments (23).

## 2 Mechanical stress properties of the TME

### 2.1 Matrix stiffness enhances NK cell motility

The TME is comprised of tumor cells, various immune cell subsets like regulatory T cells (Tregs), myeloid-derived suppressor cells (MDSCs), and tumor-associated macrophages (TAMs). It also includes components like the ECM and immunosuppressive factors such as TGF-β and PGE2 (24, 25). These elements work together to influence the function and behavior of NK cells, with substantial evidence indicating their capability to inhibit NK cell activity directly.

In solid tumors, high-stiffness ECM and mechanical stress can directly regulate immune cell function through physicochemical signals, emerging as a new intervention target for cancer immunotherapy (26, 27). Among these effects, the impact on NK cells is particularly critical: the target cell lysis function of NK cells is highly dependent on their own migratory capacity and direct cell-cell contact, making them extremely sensitive to mechanical stress signals. Unlike the unregulated inhibitory effect of stiff ECM on immune infiltration, which typically combines physical barrier obstruction with the promotion of immunosuppressive microenvironments, Piezo1-mediated mechanotransduction can “reprogram” the mechanical signal of ECM stiffness into a pro-tumor-killing response in NK cells. In fibrotic solid tumors characterized by high-stiffness matrices, the activation of the mechanosensitive ion channel Piezo1 amplifies mechanical signals and promotes dynamic assembly of F-actin, thereby remodeling the cytoskeleton and markedly facilitating the migratory capacity of NK cells, enabling their penetration of the complex three-dimensional ECM scaffold. Through Piezo1-mediated calcium influx, calmodulin-dependent kinase II (CaMKII) becomes activated (28, 29). The phosphorylated CaMKII subsequently modifies cofilin, a key regulator of F-actin depolymerization, by phosphorylation. This modification alleviates cofilin’s inhibitory effect on F-actin stability and further promotes the formation of invasive pseudopodia, which serve as a critical structural foundation for NK cells to traverse the dense ECM (30).

Within the TME, other cell types can also influence the penetration ability of NK cells. For instance, tumor cells inhibit NK cell activity by secreting TGF-β and expressing Programmed Death-Ligand 1(PD-L1) (31, 32). Tumor-Associated Fibroblasts (CAFs) inhibit the polarization of NK cell cytotoxic granules through cytokines such as Interleukin-6 (IL-6) (33). Furthermore, ECM contraction dependent on α-smooth muscle actin (α-SMA) increases local stiffness, forming physical barriers regions and impairing the infiltration capacity and functional efficacy of NK cells (34).

### 2.2 Bidirectional modulation of immune cells by mechanical stresses

Mechanical stress can exert bidirectional regulatory effects on a variety of immune cells, including NK cells, through the mechanobiology-immunology coupling mechanism. For NK cells, when matrix stiffness increases, the mechanosensitive ion channel Piezo1 is activated, which in turn promotes NK cell migration and enhances the release of gamma-interferon (IFN-γ), thereby achieving positive regulation of NK cell function (35); conversely, if the ECM is excessively cross-linked such that its porosity falls below the approximate diameter of a lymphocyte nucleus, it effectively forms a dense physical barrier. This confinement has been shown to markedly impair immune cell migration efficiency, in turn exerting a notable negative impact on NK cell infiltration and function.

Given the critical role of NK cells in cancer immunity, NK cell-based immunotherapies have continued to develop in recent years, with CAR-NK therapy standing out particularly (36). The fundamental concept of this therapy is to employ lentiviral vectors to transduce CAR structures, genetically modifying NK cells so they can recognize specific antigens (37). This alteration not only allows NK cells to accurately identify antigens like CD19 and BCMA on tumor surfaces but also enables them to detect other tumor-specific surface antigens. Through the modification of lentiviral vectors, NK cells have acquired antigen-specific recognition ability and can effectively recognize antigens on the surface of tumors. Compared with CAR-T therapy, CAR-NK therapy has a clinical advantage in that it is less likely to trigger excessive immune activation or neurotoxicity, making it safer (36, 38). In treating hematological cancers, this therapy has demonstrated a significant response rate and notable therapeutic efficacy (39).

## 3 Molecular mechanisms of mechanical stress-mediated regulation of NK cell function

### 3.1 Mechanisms of NK cells in the TME

NK cells are crucial components of innate immunity, acting as primary defenders in anti-tumor responses. These cells can detect and destroy malignant cells without prior antigenic sensitization. Their functionality is contingent upon the balanced regulation of surface receptors. For example, the NK Group 2, Member D (NKG2D) receptor can recognize stress ligands such as MHC Class I Chain-Related Proteins A/B (MICA/MICB) present on tumor cells, which prompts the release of perforin and granzyme via the Vav1-PI3K signaling pathway (40). On the other hand, killer cell immunoglobulin-like receptors (KIRs) interact with major histocompatibility complex class I (MHC-I) molecules, activating the SHP-1 signaling pathway to maintain immune tolerance (41, 42). Importantly, when tumor cells downregulate MHC-I, NK cells recognize this “missing self,” thereby boosting their cytotoxic activity (43, 44). In addition to exerting direct cytotoxic effects through NKG2D-mediated release of perforin and granzyme, NK cells also secrete tumor necrosis factor-α (TNF-α) and interferon-γ (IFN-γ) to recruit T cells and dendritic cells to the tumor site (45). This process connects innate immunity with adaptive immunity, enabling them to coordinate to suppress tumor growth (46). Furthermore, the number and activity of tumor-infiltrating NK cells (TINKs) are significantly correlated with tumor prognosis, reflecting the clinical significance of NK cell function in anti-tumor immunity (47).

Importantly, NK cells are not a homogeneous population. Human NK cells comprise at least the conventional circulating CD56^bright^ and CD56^dim^ subsets, tissue-resident NK (trNK) cells in organs such as liver and uterus, and adaptive/memory-like NK cells that arise in response to chronic viral infection (48). Current evidence suggests that mechanosensing capacity, including the expression level and functional coupling of Piezo1, may differ across these subsets; however, systematic profiling of Piezo1 in human NK populations is still lacking (49). In this review, most of the mechanistic discussion and therapeutic concepts are primarily grounded in studies using conventional, peripheral blood–derived NK cells and CAR-NK products generated from them. Explicitly recognizing NK-cell heterogeneity is essential, because subset-specific Piezo1 signaling will likely determine which NK populations derive the greatest benefit from Piezo1-centered interventions and which might require distinct strategies.

**Figure 1:**
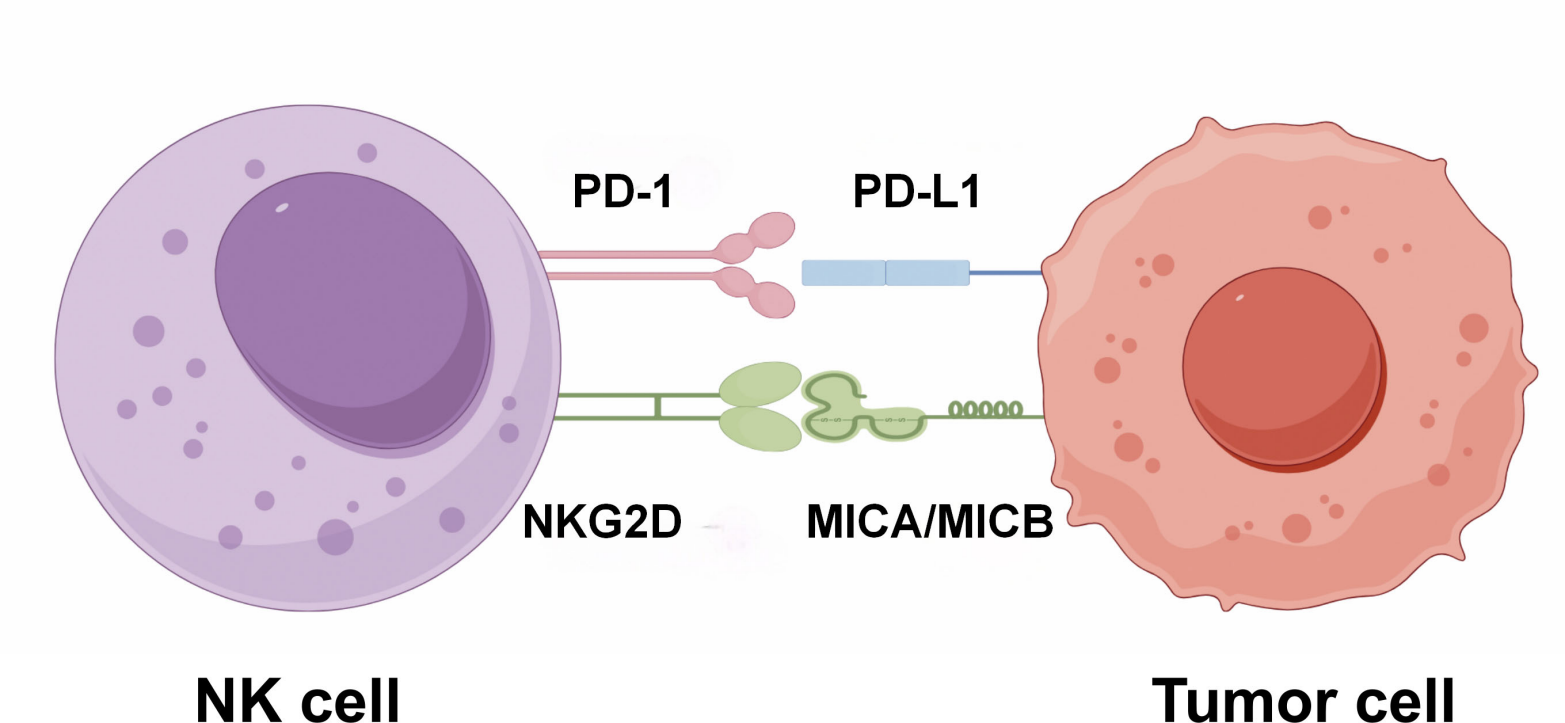
Antagonistic regulation of NK cell killing by NKG2D-MIC and PD-1/PD-L1 axes.

Within the TME, the killing efficacy of NK cells is dynamically regulated by a balance between activating and inhibitory signals (50). This regulatory mechanism is visually summarized in **Figure 1**. On one hand, the activating NKG2D receptor, which is expressed on the surface of NK cells, recognizes stress-induced ligands including MICA/MICB, that are presented on tumor cells; this recognition event directly triggers a cytotoxic response in NK cells (51). On the other hand, this cytotoxic attack can be suppressed by inhibitory checkpoint interactions. A prominent example of such interactions is the binding between PD-1 molecules on NK cells and PD-L1 proteins expressed by tumor cells (52). The final outcome of this signaling crosstalk, which is either immune attack or immune tolerance, relies on the net integration of these opposing activating and inhibitory signals. This delicate signaling balance is further complicated by other factors in the TME. For instance, immunosuppressive cytokines such as TGF-β can downregulate the expression of activating receptors like NKG2D on NK cells, and this downregulation effectively tilts the signaling balance toward tumor escape (53).

However, the infiltration and activity of NK cells in the TME are severely restricted by multiple factors. First, the dense ECM forms a mechanical barrier that reduces NK cells’ migration efficiency (54). Second, Tregs and MDSCs inhibit the activity of NK cells by secreting TGF-β and interleukin-10 (IL-10). Among these, TGF-β specifically downregulates the expression of NKG2D via Sma- and Mad-related protein 3 (SMAD3). Third, PGE2 interferes with the metabolic pathways of NK cells, thereby impairing their function (55, 56). Mechanical stress also exerts dual effects on NK cell behavior via the mechanosensitive ion channel Piezo1: acute activation of Piezo1 promotes calcium ion (Ca^2+^) influx and enhances the cytotoxicity of NK cells, while chronic mechanical stress induces NK cell depletion via the YAP/TAZ pathway and reduces perforin secretion (57).

To address the restrictions imposed by the TME on NK cells, several intervention strategies have been proposed. These include targeting TME barriers such as degrading the ECM or using TGF-β inhibitors to improve NK cell infiltration (47). Additionally, delivering IL-2 and IL-15 via the Janus kinase-signal transducer and activator of transcription 5 (JAK-STAT5) pathway can strengthen NK cell proliferation and activity while offsetting the inhibitory impacts induced by components of the tumor microenvironment. Another strategy involves employing Piezo1 agonists or ECM remodelers to optimize mechanosensitive mechanisms and boost cytotoxicity. Integrated modulation of ECM, inhibitory signaling, and mechanical stress promises to enhance NK cell efficacy in solid tumor therapy (58, 59).

### 3.2 Piezo1-mediated mechanosignaling: from channel gating to downstream pathways

**Figure 2:**
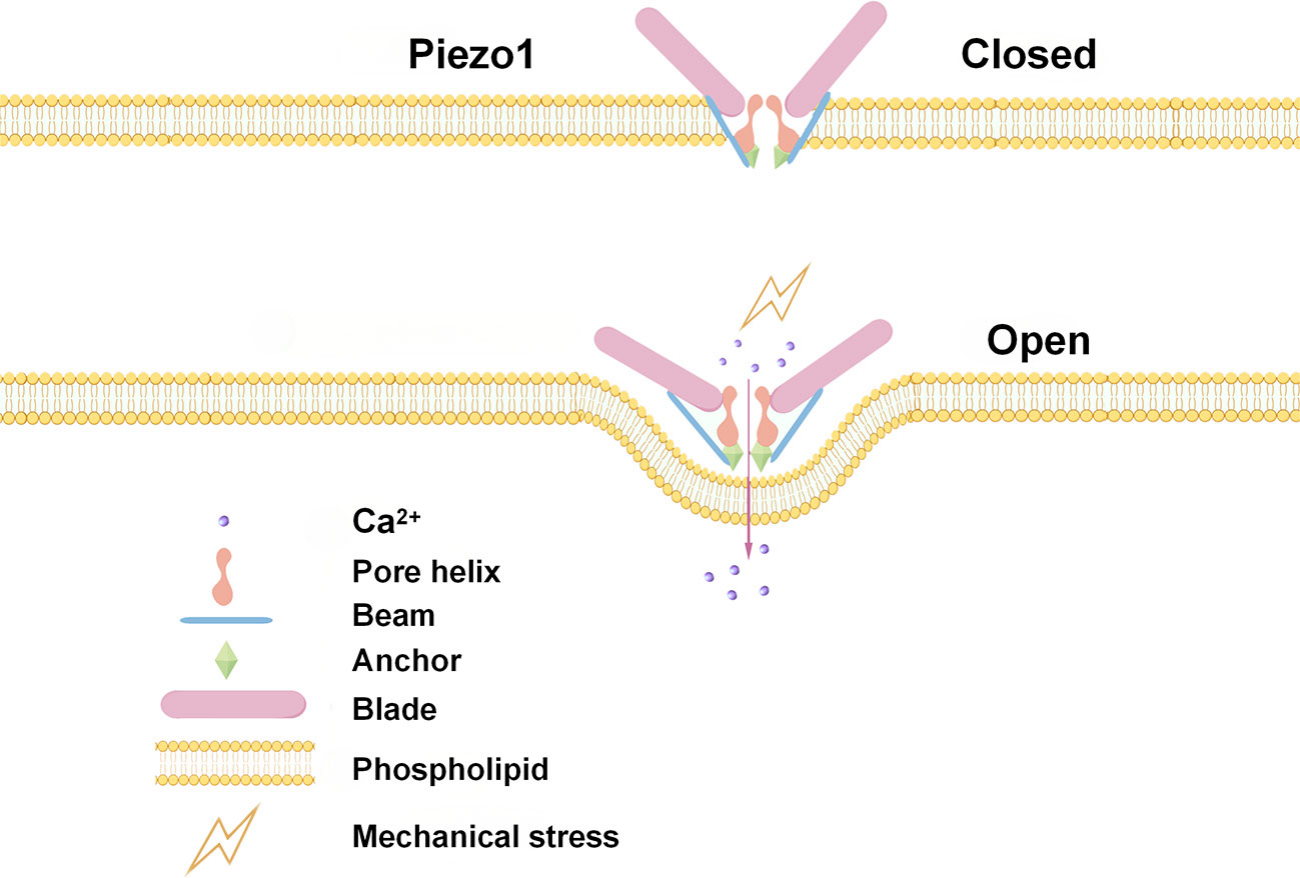
Mechanical stimulation opens Piezo channels by bending the beam to tilt the pore helix, enabling Ca^²+^ influx.

Piezo1 is a large, trimeric mechanosensitive ion channel protein localized to the plasma membrane that functions as a primary sensor of mechanical cues (60). In **Figure 2**, the unique architecture of Piezo1 is central to its ability to convert physical forces, such as membrane tension arising from ECM stiffness, into Ca^2+^ influx (61, 62). As illustrated by its structural model, the channel’s three long, blade-like peripheral domains are thought to act as force sensors. According to the force-from-lipid model of mechanogating, increased membrane tension flattens these curved blades, which in turn levers open the central ion-conducting pore (63, 64), This rapid structural rearrangement allows Ca^2+^ to flood the cytoplasm, initiating downstream mechanotransduction cascades that are critical for NK cell function.

The indispensable role of Piezo1 in NK cells has been firmly established by key functional studies. Pharmacological experiments by Solis et al. (2019) demonstrated that the specific Piezo1 agonist Yoda1 markedly enhances NK cell degranulation and cytotoxicity, an effect that persists under prolonged stimulation (65). Complementing this, Yanamandra et al. (2024) used a Piezo1 knockout model to show that its deficiency severely impairs NK cell tumor surveillance, particularly compromising stiffness-dependent cytotoxicity and infiltration into 3D matrices (14). These studies collectively confirm Piezo1 as a core mechanotransducer in NK cells. However, it is critical to recognize that these foundational studies, and thus much of our current mechanistic understanding, are primarily based on conventional peripheral blood-derived NK cells (largely the CD56^dim^ subset) and CAR-NK products generated from them (65, 66). Human NK cells are a heterogeneous population, also comprising CD56^bright^ circulating cells, trNK cells, and adaptive/memory-like NK cells (67). Whether these distinct subsets exhibit comparable Piezo1 expression levels and functional dependence, or require different modulation strategies, remains a significant and unresolved question (68). Therefore, the therapeutic concepts discussed in this review are most directly applicable to conventional NK cell-based platforms, and their relevance to other NK populations is speculative (69).

Upon activation, Piezo1-mediated Ca^2+^ influx triggers a hierarchy of downstream signaling pathways. For clarity, it is essential to distinguish between pathways directly validated in NK cells and those extrapolated from other cell types. The Mitogen-Activated Protein Kinase/Extracellular Signal-Regulated Kinase (MAPK/ERK) cascade is a downstream pathway with direct validation in NK cells. Mechanical stress activates this pathway in a stiffness-dependent manner, with robust activation in high-stiffness environments. This link is functionally significant, as phosphorylated ERK enhances perforin secretion (14, 70).

In contrast, the involvement of other signaling nodes is largely hypothesized based on studies in other cell types. For example, the role of the transcriptional co-activators YAP/TAZ is a compelling but putative model inferred from immune cells (T cells) and non-immune cells (fibroblasts), where Piezo1 activation promotes YAP/TAZ nuclear translocation. Extrapolating from these non-NK cell studies, it is reasonable to hypothesize that in response to mechanical stimulation, YAP/TAZ undergoes enhanced translocation to the nucleus in NK cells as well—which in turn may regulate target genes such as Connective Tissue Growth Factor (CTGF) and Cysteine-Rich Angiogenic Inducer 61 (CYR61), thereby potentially promoting NK cell migration (71, 72). Similarly, the proposed Ca^2+^-dependent activation of the Calmodulin-Dependent Kinase II (CaMKII)/cofilin axis, which promotes cytoskeletal remodeling by regulating F-actin stability, is a plausible mechanism for enhancing NK cell motility but has not been directly demonstrated to be a consequence of Piezo1 signaling in NK cells (inferred from T cell and macrophage studies) (32). Direct evidence for Piezo1-dependent YAP/TAZ nuclear translocation or CaMKII activation in NK cells is currently lacking, and these models require specific validation.

Ultimately, these signaling cascades are thought to converge on cytoskeletal reorganization, a critical process for both NK cell migration and cytotoxicity (73). In parallel, Piezo1 synergizes with adhesion receptors like integrins, which transmit mechanical forces from the ECM and enhance Piezo1 channel opening efficiency. This crosstalk effectively integrates mechanical and adhesive cues to co-regulate NK cell migration and killing functions (74).

### 3.3 Mechanical stress-induced functional heterogeneity of NK cells

In the TME, the effect of mechanical stress on NK cell function shows significant heterogeneity (75). The intracellular Piezo1/YAP signaling pathway is activated when NK cells are in a high-hardness ECM. This activation process acts as a ‘double-edged sword,’ reflecting to some extent the cells’ perception of and response to the mechanical environment but subsequently triggering a series of changes detrimental to the antitumor function of NK cells (76).

In ECM environments with high stiffness, the activation of Piezo1 enhances the nuclear translocation of YAP/TAZ. This activation leads to the upregulation of CYR61, thereby promoting pseudopodia formation, while CTGF guides tumor migration and further bolsters the mobility and infiltration efficiency of NK cells (77). In relatively soft matrix environments, although the activation of Piezo1 is relatively weak, it still promotes the migration and the antitumor activity of NK cells compared to conditions where functional Piezo1 is absent. Both migration and cytotoxic function are enhanced, resulting in increased antitumor activity.

Regarding intracellular regulatory mechanisms, mechanical stress modulates NK cell motility and invasiveness by inducing cytoskeletal reorganization and pseudopod formation (78). When the expression of the CYR61 protein is upregulated, it acts as a ‘flexible claw’ for NK cells, which enhances the formation of pseudopods, allowing the cells to better adapt to their surroundings and migrate more efficiently (79). CTGF, on the other hand, acts as a ‘navigator’ and promotes NK cell migration to tumor tissue. The functional heterogeneity of NK cells also relies heavily on the activation of the MAPK/ERK signaling pathway (80). It was found that inhibition of this pathway with the U0126 inhibitor significantly reduced perforin secretion in NK cells, suggesting the importance of this pathway in maintaining normal NK cell function (81). The NK cells’ functional heterogeneity is closely associated with the mechanical properties of the TME (82). Specifically, the mechanical cues generated by high-stiffness matrices and fluid shear stress may facilitate tumor immune evasion by constraining the antitumor efficacy of immune cells.

## 4 Cancer therapeutic strategies for targeting mechanosignaling

### 4.1 TME: a mechanically remodeled space

**Figure 3:**
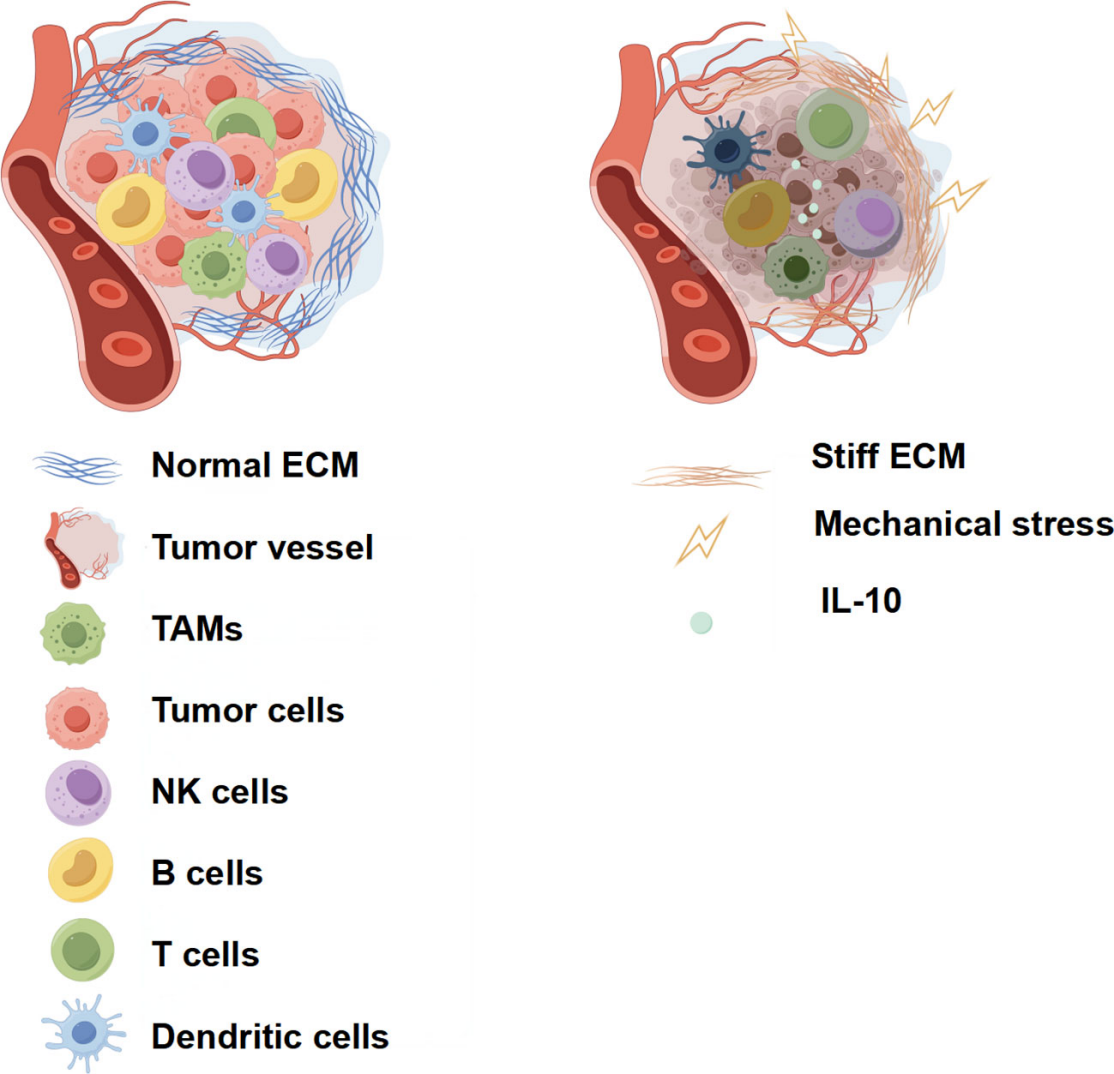
Contrasting effects of normal (left) and stiff (right) ECM on tumor immune infiltration.

The properties of the ECM exert a profound and contrasting influence on tumor immune infiltration, a dichotomy clearly illustrated in **Figure 3** (27). A loose, physiologically normal ECM, depicted on the left, features sparse collagen fibers and large pores, creating a permissive environment that facilitates the free migration and surveillance activities of immune cells like NK and T cells. In stark contrast, the tumor-associated ECM, shown on the right, becomes a formidable barrier that suppresses antitumor responses through at least two distinct mechanisms (1). First, excessive deposition and crosslinking of collagen creates a dense fibrous barrier that physically impedes immune cell migration, as represented by the trapped NK cell in the illustration; this physical obstruction has been shown to reduce NK cell migration efficiency in preclinical models (83). Second, this stiffened matrix promotes an immunosuppressive milieu by enhancing the secretion of inhibitory factors, such as TGF-β from cancer-associated fibroblasts and IL-10 from tumor-associated macrophages (TAMs), which directly impair the cytotoxic function of nearby immune cells (53). Importantly, this ECM remodeling is not a passive consequence of tumor growth but can represent an active adaptive immune evasion strategy. Tumors may dynamically modulate their mechanical properties to escape Piezo1-dependent immunosurveillance. For instance, while high global stiffness can potentiate NK cell function, cancer cells can exploit matrix plasticity to create migration-inhibiting micro-architectures or even generate localized “soft” niches by secreting matrix-degrading enzymes (84). Such mechanically heterogeneous niches could dampen Piezo1 signaling in infiltrating NK cells, creating sanctuaries for tumor survival and thereby constituting a form of adaptive resistance to mechanical immunotherapy.

### 4.2 Piezo1-targeted intervention and CAR-NK combination strategy

Although CAR-NK therapy has achieved remarkable results in hematological malignancies, it still faces many challenges when applied to solid tumors (85). On one hand, the solid TME is more complex: the dense ECM barrier seriously hinders the infiltrative process of NK cells into the tumor core, and adverse metabolic environments such as hypoxia and acidity further inhibit the activity of NK cells (86, 87). Conversely, in the immunosuppressive microenvironment, CAFs release cytokines like TGF-β and IL-6 that directly suppress NK cell function. This results in a 40% reduction of CD16 molecule expression on the surface of NK cells. Additionally, the hypoxic conditions (oxygen partial pressure PO_2_ <10 mmHg) can decrease the killing activity of NK cells by 55% (88, 89). Additionally, there are technical challenges in both the preparation and application of NK cells. Expanding autologous NK cells is a complex process, often involving 5 to 10 cycles of *in vitro* expansion (90). Allogeneic NK cells introduce risks like potential rejection. Moreover, the *in vivo* survival of NK cells is limited, with up to 80% being cleared within 72 hours after intravenous injection (91). These challenges related to preparation technologies can significantly impact the therapeutic efficacy (92).

The therapeutic limitations of CAR-NK cells in solid tumors are primarily due to the combined effects of the physical barriers of the TME and its immunosuppressive conditions (93). Targeting the mechanosensitive ion channel Piezo1 offers a promising solution to these challenges. By mediating mechanical signal transduction, Piezo1 can enhance the ability of NK cells to penetrate the ECM and help them overcome the inhibitory effects of CAFs. Therefore, the combined strategy of Piezo1 modulation with CAR-NK therapy represents a potential direction for the treatment of solid tumors. Mechanical signals serve as a central regulator in governing the function of NK cells (94). As a key mechanosensitive ion channel, Piezo1 enables NK cells to sense external mechanical stress and converts this mechanical stress into an electrochemical signal by mediating the influx of calcium ions, denoted as Ca^2+^ (95). This process plays a critical role in sustaining the cytotoxicity and migratory capacity of NK cells (96). Activation of Piezo1 triggers Ca^2+^ influx, which enhances the effector functions of NK cells, including increased release of perforin and granzyme as well as cytoskeletal reorganization (97). This enhancement further improves the ability of NK cells to penetrate dense three-dimensional matrices and kill tumor cells (65). In contrast, inhibition of Piezo1 using agents such as Grammostola spatulata mechanotoxin-4 (GsMTx4) reduces the migration distance of NK cells in the ECM, which confirms that the function of NK cells is highly dependent on mechanotransduction (98, 99). Notably, Piezo1 activation consistently bolsters NK cell antitumor activity in stiff TME. In the context of highly fibrotic lesions, where ECM stiffness is significantly elevated, Piezo1-mediated mechanotransduction actively enhances NK cell infiltration through the matrix and preserves cytolytic function (100, 101). This effect can be further amplified by combining it with ECM-remodeling strategies, such as targeted collagen degradation, to reduce physical barriers. This finding clearly demonstrates the determinant role of TME stiffness in Piezo1-mediated regulation of NK cell fate. Accordingly, therapeutic strategies can be designed in two aspects: first, utilizing Piezo1 agonists to enhance the infiltration capacity and cytotoxic efficacy of NK cells in tumors with lower stiffness (102); second, remodeling the ECM to soften the TME, thereby improving the infiltration efficiency of immune cells (103, 104). Specifically, remodeling of the ECM can be achieved through two approaches: inhibiting fibroblast activity or degrading hyaluronic acid.

This mechanical barrier represents a major obstacle for adoptive cell therapies, including CAR-NK therapies (105). While CAR-NK therapy exhibits significant safety and efficacy in hematological malignancies such as those targeting CD19 or BCMA, its success in solid tumors remains limited. The primary challenges include the inadequate infiltration of CAR-NK cells into the dense tumor stroma, suppression by cancer-associated fibroblasts (CAFs), and the impact of immunosuppressive cytokines like TGF-β. Additionally, there is the induction of exhausted phenotypes caused by checkpoint molecules such as PD-L1 (106). Therefore, combining Piezo1-targeted intervention with CAR-NK therapy represents a rational strategy, although it is still in the preclinical stage (107). Within this synergistic effect, a major concept is to use Piezo1 agonists to provide mechanical “boosting” for CAR-NK, thereby enhancing their ability to navigate and penetrate solid tumors (14, 65). Transient modulation of Piezo1–YAP/TAZ signaling has been put forward as a strategy to reduce or delay effector-cell exhaustion, primarily based on findings from T cell and other immune subset research. However, whether a comparable mechanism functions in NK cells remains undetermined, and this idea should be regarded as a hypothesis rather than an established biological fact.

Translating this combination strategy into clinical practice requires overcoming numerous challenges, underscoring its speculative nature (108). First, systemic administration of Piezo1 agonists such as Yoda1 poses significant toxicity risks (109). Off-target activation of Piezo1 in non-immune cells such as red blood cells and endothelial cells may lead to hemolysis or cardiovascular complications, thereby limiting the therapeutic window of these agonists (110). Second, the development of Piezo1 inhibitors such as GsMTx4 faces challenges related to delivery and potential immunogenicity (111, 112). Additionally, although many phase I/II clinical trials are evaluating the use of CAR-NK for solid tumor treatment, including therapies targeting mesothelin in pancreatic cancer or HER2 in breast cancer, these trials still grapple with issues of infiltration and persistence, which are precisely the problems that mechanical modulation aims to address (113, 114). To date, there have been no clinical trials investigating the combination therapy of Piezo1 targeting and CAR-NK (115, 116), primarily because most specific small-molecule inhibitors or agonists targeting Piezo1 remain in the preclinical stage and no mature candidate drugs have entered human clinical trials. Without mature targeted drugs, the aforementioned translational bottlenecks have not been resolved, so the combination therapy remains at the theoretical stage. In the future, advancements in Piezo1-targeted drug research and development hold promise. Strategies such as enhancing the specificity of small-molecule inhibitors or agonists through structural optimization and minimizing off-target toxicity, alongside using nanodelivery systems to achieve precise drug enrichment in the TME, are key areas of focus. Additionally, thoroughly investigating the synergistic mechanisms between Piezo1 targeting and CAR-NK cells in preclinical studies is anticipated to gradually overcome current translational barriers. These efforts aim to pave the way for subsequent clinical trials of their combination, advancing this potential therapeutic strategy from theoretical exploration to clinical application, and offering a new direction for the treatment of solid tumors (117, 118). To fully understand the potential and complexity of Piezo1 as a therapeutic target, it is essential to recognize that its functions extend far beyond the regulation of NK cells. In fact, ECM stiffness and Piezo1-mediated mechanotransduction play pivotal roles in the progression of various tumors. **Table 1** systematically summarizes the cross-cancer evidence, illustrating how Piezo1 influences angiogenesis in hepatocellular carcinoma, stromal remodeling in pancreatic cancer, and chemoresistance in ovarian cancer. These diverse functions not only highlight the role of Piezo1 as a central hub in cancer mechanobiology but also lay the foundation for subsequent discussions on its therapeutic challenges and evidence hierarchy.

**Table 1:** Role of ECM stiffness and Piezo1 in modulating tumor progression and microenvironment.

Tumor type	Clinical ECM stiffness (kPa)	*In vitro* model stiffness (kPa)	Piezo-mediated response (mainly Piezo1)	Key experimental findings	References
Hepatocellular Carcinoma (HCC)	Primary: 10–25; Highly invasive: ≥20; ICC, control: >25	≥15 kPa (FN-gel, invasive mimic); 15 kPa (collagen, primary mimic); 5 kPa (collagen, normal control)	Activates Integrin β1/Piezo1/Ca^2+^/HIF-1α pathway; Promotes Vascular Endothelial Growth Factor (VEGF) (pro-angiogenesis); Enhances cell proliferation	Piezo1 activation drives VEGF-mediated angiogenesis. Synergistic antitumor effect: Matrix softening + Piezo1 blockade.	(119, 120)
Pancreatic Cancer(PDAC)	Normal: 0.15–2; Primary: 1–7; Advanced: >30	30 kPa (polyacrylamide, advanced mimic); <10 kPa (Matrigel, normal control); 20 kPa (collagen, primary mimic)	Mediates Piezo1/Ca^2+^/PYK2 → TAM differentiation; Ultrasound-microbubble → apoptosis; Enhances autophagy (LC3-II)	Ultrasound-microbubble induces Piezo1-dependent apoptosis. Matrix stiffness enhances stemness via augmented autophagy.	(121, 122)
Breast Cancer(BC)	Normal: 2–5; Primary (ductal): 8–20; Lung metastatic: 12–25	3–8 kPa (collagen-Transwell, metastasis mimic); 1.2/3.5 kPa (AFM, cell stiffness)	Regulates actin reorganization; Activated Piezo1 → higher cell stiffness; Inhibits invasion (confined microenvironment)	Piezo1 KD bidirectionally regulates migration. Suppresses invasion by reducing pseudopods and Matrix Metalloproteinase (MMP) expression.	(123)
Ovarian Cancer (OvCa)	Normal stroma: 5–8; SKOV3: 11.5; OVCAR3: 7.8	15–16 kPa (FN-gel, high-progression mimic); 5 kPa (FN-gel, normal control	Rewires lipid metabolism ↑Fatty Acid Synthase (FASN); High stiffness → Piezo1 membrane ↓ → chemoresistance; Regulates Ca^2+^- Protein Kinase B (AKT) pathway	Matrix stiffness rewires lipid metabolism (FASN upregulation). Promotes chemoresistance via membrane cholesterol accumulation.	(124)
Acute Myeloid Leukemia (AML)	Healthy bone marrow: 0.1–0.3; Leukemic: <0.5	0.4 kPa (alginate, leukemic mimic); 0.2 kPa (alginate, healthy control)	Regulates NK cell mechano-sensing/killing; Promotes NK polarization + perforin/granzyme B; Low stiffness → Piezo1 signaling ↓	Piezo1 senses target stiffness to regulate NK killing efficiency. Piezo1 inhibition impairs NK infiltration and cytotoxicity.	(14)

### 4.3 Evidence hierarchy in Piezo1-NK biology: findings, hypotheses, and therapeutic concepts

Given the rapid expansion of Piezo1 research in mechanobiology and NK cell immunology, current findings span a spectrum of evidential strength. It is essential to distinguish well-supported observations from nascent hypotheses and forward-looking therapeutic ideas.

Several findings have now been directly demonstrated in NK cells or reproduced across multiple experimental systems. Piezo1 operates as a bona fide mechanosensor in NK cells, converting extracellular matrix stiffness and applied mechanical force into intracellular Ca^2+^ influx and downstream signaling (14). Acute activation of Piezo1, achieved either by increases in matrix stiffness or by short-term exposure to the small-molecule agonist Yoda1, has been shown to enhance NK cell degranulation, perforin/granzyme-mediated cytotoxicity, and three-dimensional matrix infiltration *in vitro* and in preclinical models. Complementary loss-of-function experiments using genetic ablation or pharmacologic inhibitors such as GsMTx4 attenuate these stiffness-dependent NK effector functions and reduce tissue penetration in model systems. Mechanistic work implicates MAPK/ERK pathway engagement as one mediator of mechanically driven cytotoxic responses.

Beyond these experimentally validated results lie a set of plausible but incompletely validated mechanistic propositions. While short-term Piezo1 activation augments NK activity, whether sustained or repeated Piezo1 stimulation can preserve NK functionality without inducing exhaustion, characterized by upregulation of PD-1, TIM-3, or other dysfunction markers, remains unresolved and requires systematic long-term studies. Similarly, the involvement of the Piezo1–YAP/TAZ axis in NK cell fate decisions and exhaustion is an attractive model drawn in part from T cell and non-immune studies (125). However, direct demonstration in NK cells, encompassing YAP/TAZ nuclear translocation, target gene regulation, or functional rescue experiments, has not yet been reported. Proposed molecular crosstalks, including putative YAP/TAZ amplification of MAPK/ERK signaling or promoter-level regulation of MEK1 in NK cells, likewise remain inferential and should be tested using NK-specific molecular and transcriptional assays, longitudinal stimulation protocols, and *in vivo* validation.

At the translational frontier are several speculative therapeutic concepts that, while conceptually appealing, currently lack robust preclinical efficacy or safety data. One such concept involves the co-modulation of CAR-NK platforms with Piezo1-targeted approaches to reduce neurotoxicity. Although CAR-NK therapies are generally regarded as safer than CAR-T, there is no clinical evidence that Piezo1 engagement further lowers neurotoxicity rates to specific thresholds (18). The idea that temporally precise modulation of Piezo1 activity could be used to overcome fibrotic or stiffened tumor microenvironments is theoretically attractive but experimentally untested. Similarly, strategies for tumor-localized Piezo1 targeting via nanoparticle-mediated local delivery aim to enhance NK function while minimizing off-target activation in endothelial cells or erythrocytes, yet pharmacokinetic, biodistribution, and toxicity profiles for such approaches are currently lacking. This tiered appraisal, distinguishing validated mechanisms, high-priority hypotheses, and speculative therapeutic directions, collectively provides a clearer map of current knowledge and highlights the experimental and translational gaps that should guide future work.

### 4.4 Limitations, controversies, and open questions in Piezo1–NK mechanobiology

The translational potential of Piezo1-targeted NK cell immunotherapy is significantly constrained by inherent biological limitations and unaddressed safety concerns, primarily stemming from Piezo1’s ubiquitous tissue expression (63). Unlike immune-restricted molecules, Piezo1 fulfills essential physiological roles in non-immune tissues: it regulates erythrocyte volume homeostasis (linked to hemolytic anemia) and mediates shear stress sensing in vascular endothelial cells. These roles translate to substantial off-target risks: agonists like Yoda1 induce erythrocyte lysis and can disrupt vascular integrity via cross-activation of TRPV4 channels. Compounding this, current tools (Yoda1, GsMTx4) lack cell-type selectivity (126). Systemic administration would indiscriminately affect numerous cell types, and preclinical NK cell studies have rarely assessed these off-target effects, severely limiting the translational outlook.

Beyond safety, the field suffers from insufficient mechanistic depth, particularly *in vivo*. Most conclusions derive from short-term *in vitro* experiments that fail to model the complexity of the TME (4). Critical questions remain: While acute stimulation enhances cytotoxicity, no study has validated whether sustained activation avoids inducing exhaustion (e.g., up-regulation of PD-1, TIM-3) in fibrotic TMEs. The proposed Piezo1-YAP/TAZ axis in NK cell exhaustion exemplifies this knowledge gap. While YAP/TAZ is implicated in T-cell exhaustion, its role in NK cells is not only unproven but also potentially contradictory. Notably, recent findings indicate that human NK cells exclusively express TAZ (not YAP), and that cytoplasmic TAZ actually constrains NK cell cytotoxicity, contrasting sharply with its pro-exhaustion role in T cells (12). This highlights the danger of direct extrapolation between immune cell types and underscores that the Piezo1-YAP/TAZ link in NK cells remains highly speculative.

A more significant challenge, however, is the pro-tumorigenic role of Piezo1 itself in cancer cells (127). The assumption that Piezo1 modulation will selectively benefit antitumor immunity is flawed, as Piezo1 is frequently upregulated in various cancer types, where its activation promotes proliferation, invasion, and chemoresistance (128). For example, in glioma and breast cancer, Piezo1 channels drive cellular aggression by activating pathways like MAPK/ERK. This creates a therapeutic paradox: a systemic Piezo1 agonist intended to boost NK cell function could simultaneously accelerate tumor progression. This dual functionality necessitates the development of strategies that can spatially and temporally confine Piezo1 modulation to immune effector cells, for instance, by engineering NK cells with conditionally active Piezo1 variants or using nanoparticle-based delivery systems that specifically target NK cells.

These foundational limitations are compounded by technical and translational bottlenecks. Piezo1’s structure hinders the design of selective modulators, and delivery systems have yet to achieve tumor-localized delivery to avoid systemic toxicity (129, 130). Practical challenges in clinical implementation—such as impacts on CAR-NK engineering protocols, the stability of genetically modified Piezo1 variants during lentiviral transduction, compromised ex vivo expansion efficiency of NK cells after Piezo1 activation due to enhanced metabolic demand or cytoskeletal remodeling, Good Manufacturing Practice (GMP)-compliant manufacturing of Piezo1-targeted reagents, scale-up production of NK cell-specific nanoparticles, and the absence of long-term toxicity assessments in preclinical models. These challenges remain unaddressed. No Piezo1 modulator has entered Phase I trials, and pharmacokinetic deficits such as Yoda1’s short half-life further complicate clinical translation.

Addressing these gaps requires a strategic roadmap. First, it is imperative to systematically profile Piezo1 expression and function across diverse human NK cell subsets (conventional, trNK, adaptive) to determine which populations are most amenable to this strategy. This will resolve the critical uncertainty about which cells would benefit from Piezo1-centered interventions. Second, future work must focus on developing NK cell-specific delivery systems to mitigate off-target toxicity and resolve the therapeutic paradox. Third, the functional consequences of the Piezo1-TAZ axis must be validated in NK cell-specific *in vivo* models (131). Without addressing these fundamental biological and technical hurdles, the translational roadmap for Piezo1-NK therapy remains overly optimistic and disconnected from clinical reality (132).

## 5 Conclusion

In studies aimed at enhancing the efficacy of cancer immunotherapy, the physical stiffness of the TME is regarded as a major barrier. This issue not only impairs the antitumor function of NK cells but also limits the therapeutic efficacy of CAR-NK. Piezo1 is a mechanosensitive ion channel that serves as a regulatory hub, playing a crucial role in various cellular processes, transforms mechanical signals within the TME into intracellular Ca^2+^ signals. This action unidirectionally potentiates the antitumor activity of both NK cells and CAR-NK cells. Targeting Piezo1 provides a new strategy for current immunotherapies. This approach specifically addresses the physical barrier that hinders immune cell infiltration into solid tumors.

However, the clinical translation of Piezo1 modulators still faces significant bottlenecks, including their potential risks and limitations. Firstly, the regulation of Piezo1 signaling is highly context-dependent. In the dense fibrotic TME, prolonged activation of Piezo1 supports NK cell efficacy by enhancing their ability to penetrate the matrix. This highlights Piezo1 as a promising target for overcoming the physical barriers posed by solid tumors. Second, there is insufficient targeting specificity: current Piezo1 pharmacological modulators, with Yoda1 as a representative, lack cell specificity. Since Piezo1 is widely expressed in normal tissues in the body, systemic administration may cause non-specific effects and easily trigger off-target effects such as vascular dysfunction, which makes it difficult to define a safe and effective therapeutic window. Additionally, the lack of precision in Piezo1 modulation (e.g., timing and dosage) and tumor heterogeneity further hinder clinical translation; moreover, long-term application of modulators may induce drug resistance, exacerbating these translational challenges. Future research should focus on three primary directions. First, develop cell-specific delivery systems to precisely deliver Piezo1 modulators via nanotechnology or engineered materials, thereby enhancing therapeutic efficacy and reducing systemic toxicity. Second, formulate personalized treatment regimens by incorporating tumor type, TME stiffness, and Piezo1 expression. This enables the targeted adjustment of modulator dosage and timing to prevent the exhaustion of NK cells induced by TGF-β and hypoxia, while maintaining their robust activity. Third, explore combination strategies to overcome drug resistance, such as combining transient Piezo1 agonists with CAR-NK therapy and targeting drug resistance-related pathways to prevent adaptive escape. Future investigations should prioritize the systematic mapping of Piezo1 expression and function across heterogeneous NK subsets, which will inform the rational design of next-generation CAR-NK therapies. By implementing these strategies, we aim to more safely utilize the Piezo1-mediated mechanotransduction mechanism, effectively address the physical barriers posed by solid tumors, and establish a robust foundation for the clinical application of mechanical immunotherapy. This approach has the potential to significantly enhance CAR-NK therapy in the treatment of solid tumors.

## Glossary

3D: three-dimensional
NK: Natural Killer
TME: Tumor microenvironment
ECM: Extracellular Matrix
TGF-β: Transforming Growth Factor-β
PGE2: Prostaglandin E2
Ca^2+^: Calcium Ion
Piezo1: Piezo-type mechanosensitive ion channel component 1
YAP/TAZ: Yes-associated protein/Transcriptional co-activator with PDZ-binding motif
CAR-T: Chimeric Antigen Receptor T-cell Therapy
CAR-NK: Chimeric Antigen Receptor Natural Killer Cell Therapy
TCR: T Cell Receptor
CaMKII: Calmodulin-Dependent Kinase II
PD-L1: Programmed Death-Ligand 1
CAFs: Tumor-Associated Fibroblasts
IL-6: Interleukin-6
α-SMA: α-Smooth Muscle Actin
IFN-γ: Gamma-Interferon
MDSCs: Myeloid-Derived Suppressor Cells
TAMs: Tumor-Associated Macrophages
Tregs: Regulatory T Cells
NKG2D: Natural Killer Group 2, Member D
MICA/MICB: MHC Class I Chain-Related Proteins A/B
KIRs: Killer Cell Immunoglobulin-Like Receptors
MHC-I: Major Histocompatibility Complex Class I
TNF-α: Tumor Necrosis Factor-α
TINKs: Tumor-Infiltrating NK Cells
IL-10: Interleukin-10
SMAD3: Sma- and Mad-Related Protein 3
JAK-STAT5: Janus Kinase-Signal Transducer and Activator of Transcription 5
MAPK: Mitogen-Activated Protein Kinase
AKT: Protein Kinase B
ERK: Extracellular Signal-Regulated Kinase
CTGF: Connective Tissue Growth Factor
CYR61: Cysteine-Rich Angiogenic Inducer 61
NO: Nitric Oxide
ROS: Reactive Oxygen Species
PO2: Partial Pressure of Oxygen
GsMTx4: Grammostola spatulata Mechanotoxin-4
HCC: Hepatocellular Carcinoma
OvCa: Ovarian Cancer
PDAC: Pancreatic Ductal Adenocarcinoma
BC: Breast Cancer
AML: Acute Myeloid Leukemia
FASN: Fatty Acid Synthase
GMP: Good Manufacturing Practice
VEGF: Vascular Endothelial Growth Factor
MMP: Matrix Metalloproteinase.
